# Investigation on the Thermoforming of Pmsq-Hdpe for the Manufacture of a NACA Profile of Small Dimensions

**DOI:** 10.3390/polym13101622

**Published:** 2021-05-17

**Authors:** Erchiqui Fouad, Abdessamad Baatti, Karima Ben Hamou, Hamid Kaddami, Mhamed Souli, Abdellatif Imad

**Affiliations:** 1 École de Génie, Université du Québec en Abitibi-Témiscamingue, 445, Boulevard de l’Université, Rouyn-Noranda, QC J9X 5E4, Canada; Abdessamad.Baatti@uqat.ca (A.B.); Karima.BenHamou@uqat.ca (K.B.H.); 2 Faculté des Sciences et Techniques, Université Caddi Ayad, Marrakech 40000, Morocco; h.kaddami@uca.ma; 3 Unité de Mécanique de Lille, Université de Lille, UML, Joseph Boussinesq, F5900 Lille, France; m-hamed.souli@univ-lille.fr (M.S.); abdellatif.imad@polytech-lille.fr (A.I.)

**Keywords:** nanocomposite HDPE-PMSQ, NACA profile, thermoforming

## Abstract

Unmanned aerial vehicles (UAVs) or drones are attracting increasing interest in the aviation industry, both for military and civilian applications. The materials used so far in the manufacture of UAVs are wood, plastic, aluminum and carbon fiber. In this regard, a new family of high-density polyethylene (HDPE) nanocomposites reinforced with polymethylsilsesquioxane nanoparticles (PMSQ), with mechanical performances significantly superior to those of pure HPDE, has been prepared by a fusion-combination process. Their viscoelastic properties were determined by oscillatory shear tests and their viscoelastic behavior characterized by the Lodge integral model. Then, the Lagrangian formulation and the membrane theory assumption were used in the explicit implementation of the dynamic finite element formulation. For the forming phase, we considered the thermodynamic approach to express the external work in terms of closed volume. In terms of von Mises stress distribution and thickness in the blade, the results indicate that HDPE-PMSQ behaves like virgin HDPE. Furthermore, its materials, for all intents and purposes, require the same amount of energy to form as HDPE.

## 1. Introduction

A polymer nanocomposite may be defined as a polymer filled with particles having dimensions in the nanometer range (from 1 to 100 nm). These nanoparticles have been found to improve the stiffness, strength and thermal properties of polymers significantly at very low concentration compared to conventional fillers. This has opened up possibilities for producing high-performance composites. Most often, clays [1], carbon nanotubes [2] and nanosilica [3] have been the preferred fillers. However, a new class of hydrophobic nanofillers, polymethylsilsesquioxane (PMSQ) nanoparticles, have been synthesized [4] and are promising for the development of high thermal and mechanical performances nanostructured materials.

Typically, the PMSQ is a macromolecule, with methyl groups in its backbone, belonging to the class of double-chain polymers. Because of this structure, and compared with single-chain polymers, PMSQ polymer has a much higher resistance to thermal and chemical degradation as well as excellent mechanical strength [5]. The PMSQ has been widely developed for applications in industrial raw materials such as resins, oils and rubbers [6]. Baatti et al. [4] have successfully manufactured a ladder structure of PMSQ nanoparticles with a size of 15–20 nm, a hydrophobic surface and a good thermal stability.

Many applications of polysilsesquioxane (PSQ) in general, including PMSQ, are found in different industries because of the superior properties of these materials. This includes hybrid materials for coating [7,8], storage of energy [9,10,11], drug carriers and their liberation control [12,13], environment protection [14,15]. However, at the best of our knowledge, the potential for the development of PMSQ-based functional nanocomposites for the manufacture of unmanned aerial vehicle (UAV) blades by thermoforming, has not yet been elucidated in the literature. In this regard, a new family of high-density polyethylene (HDPE) nanocomposites reinforced with polymethylsilsesquioxane nanoparticles (PMSQ), with mechanical performances significantly superior to those of pure HPDE, has been prepared by a fusion-combination process. Their viscoelastic properties were determined by oscillatory shear tests and their viscoelastic behavior characterized by the Lodge integral model. Then, the Lagrangian formulation and the membrane theory assumption are used in the explicit implementation of the dynamic finite element formulation. For the forming phase, we considered the thermodynamic approach to express the external work in terms of closed volume

This paper is aims to study the potential use of a composite based on high density polyethylene and polymethylsilsesquioxane nanoparticles (PMSQ-HDPE) for the manufacture of small UAV blades.

## 2. Polymethylsilsesquioxane High-Density Polyethylene (PMSQ-HDPE) Composite and Characterization

### 2.1. PMSQ-HDPE Composites Elaboration

This work is part of the work carried out on the development of a nano-composite family based on PMSQ (polymethylsilsesuiqoxane), with a purity of 99%, and a high-density polyethylene matrix, HDPE [4,16]. The following are the characteristics of HDPE and PMSQ used in the manufacture of HDPE-PMSQ nanocomposites:

− The PMSQ nanoparticles has a size of 15 to 20 nm, a hydrophobic surface and good thermal stability. These nanoparticles have a very large surface area (39.721 m^2^/g^−1^) offering a very good ability to interact specifically with other hydrophobic surfaces [4].
− HDPE Hival-500354 with a melt flow index of 0.03 g min^−1^ (ASTM D1505) and a density of 0.954 g cm^−3^ (ASTM D1238) was supplied by IDES Prospector North America.

The method for the development of HDPE-PMSQ nanocomposites was based on a fusion mixing process. The procedure used to make the samples was the following: HDPE was previously dried at 363 K in a Conair dryer and the PMSQ nanoparticles were suspended in cyclohexane with one of the following concentrations (% per mass): 0.5 and 1% using an Ultra-Turrax system at 20,000 rpm and sonication for 30 min. All blends were prepared by melt mixing using a co-rotating Coperion ZSK25WLE twin-screw extruder with a segmented screw and barrel. The screw length was set at 1040 mm with a screw diameter of 25 mm and a L/D ratio of 41.6. The temperature profile of the extruder from feeding to the die was 353/468/468/473/478/478 K, the rotation speed of the twin-screw was set at 80 rpm, and the throughput was 7 kg/h^−1^. The PMSQ suspension was pumped into the melting zone. The throughput, screw speed and cyclohexane injection rate were adapted to the experiment in order to produce the following nanocomposites: HDPE-0.5% and HDPE-1%. All blends were extruded in the form of threads (4 mm diameter), dipped into a cold-water bath, dried by compressed air and then granulated by a Scheer Bay BT25 strand pelletizer granulator. After extrusion, the compounded pellets were dried. Dynamic mechanical analysis specimens were then molded according to American Society for Testing and Materials (ASTM) Standard D570 using an Arburg Allrounder 370A66 injection press. Then, the nanocomposites were characterized (Fourier transformation in the infrared, transmission electron microscopy, differential scanning calorimetry, scanning electron microscopy, mechanical tests, thermophysical characterization) [4,16]. The mechanical and thermomechanical properties obtained from HDPE-PMSQ nanocomposites have been related to the barrier effect of PMSQ nanoparticles.

The results of the analysis of PMSQ nanoparticle properties are given in Table 1. The measured apparent contact angle is that of a hydrophobic surface (148 ± 3). PMSQ nanoparticles are relatively dense (1.42 g/cm^3^) and have a good thermal stability.

### 2.2. Mechanical Properties of PMSQ-HDPE Nanocomposites

Concerning mechanical properties of the material, the elastic modulus, yield stress, and elongation at break of the neat HDPE and its nanocomposites are shown in Table 2. When compared to neat polymer resin (HDPE), the elastic modulus of HDPE nanocomposites is shown to be slightly improved. The same pattern was observed for the yield stress and approximately for the elongation at break. This enhancement can be attributed to the fact that PMSQ nanoparticles act as stress transfer agents and resists breakage, giving better strength to the nanocomposites.

### 2.3. Thermal Analysis of HDPE–PMSQ Nanocomposites

PMSQ is a macromolecule, with a methyl group on its surface, belonging to the class of double chain polymers. Due to this structure, and compared to single chain polymers, PMSQ polymer has much higher resistance to thermal degradation [16]. For this purpose, we performed a thermal analysis using differential scanning calorimetry (DSC). In Table 3, melting (T_m_) and crystallinity (T_c_) temperatures, enthalpy (DH) and relative crystallinity (X_c_) values during the heating and cooling stages are reported. It is observed that crystallization temperatures and crystallinity are slightly affected by the presence of PMSQ nanoparticles, without a clear dependency on the nanofiller content. Thus, X_c_ of HDPE-1% nanocomposite is about 1.5% higher than that of the neat matrix. This probably means that PMSQ nanoparticles having higher surface area could play some nucleating effect on the HDPE matrix. However, further investigations are needed to reach a better comprehension of the crystallization phenomena in these nanocomposite systems.

### 2.4. Morphology of HDPE–PMSQ Nanocomposites

**Figure 1:**
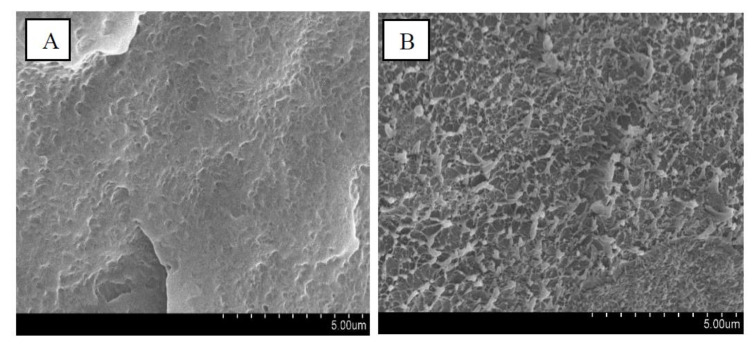
Scanning electron microscopy (SEM) micrographs of cryofractured surfaces of: (**A**) HDPE and (**B**) HDPE-1% nanocomposite elaborated with cyclohexane assisted extrusion process.

For this end, we refer to the morphology comparison between HDPE matrix and HDPE-1% nanocomposite elaborate with a cyclohexane-assisted extrusion process. It is however to be noted that some experimental observations, such as SEM, evidence very good physical contact between polymers and nanoparticles. Indeed, strong interactions between PMSQ and HDPE were evidenced by scanning electron microscopy (SEM) results and shown in Figure 1A. The nature of the fractured surface morphology of HDPE-1% nanocomposite was different compared to HDPE matrix. It can be seen that the cryo-fractured surface of pure HDPE (Figure 1A) is slightly flat and smooth. By contrast, the occurrence of stress whitening is evident in the cryo-fractured surfaces of the nanocomposites (Figure 1B). The stress whitening is related to the ductile response of the HDPE-PMSQ blends. This can reflect the good interaction between the PMSQ nanoparticles and the HDPE matrix.

### 2.5. Rheological Properties of the HDPE-PMSQ Nanocomposites

**Figure 2:**
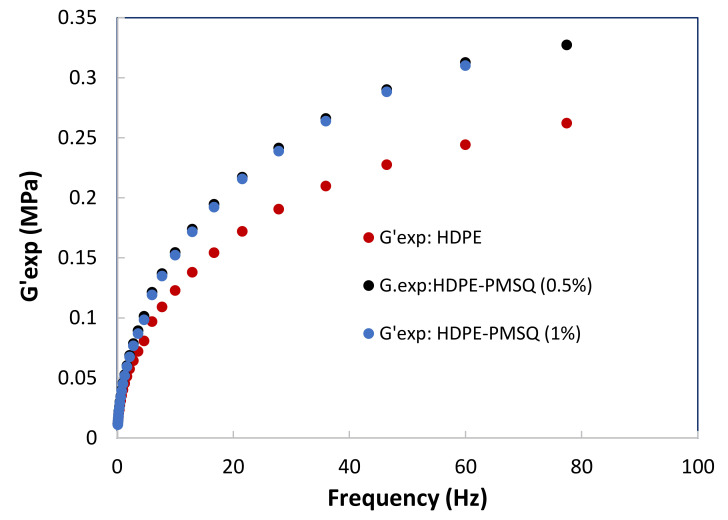
Experimental results of the storage moduli (G’) as a function of the PMSQ nanoparticles content.

**Figure 3:**
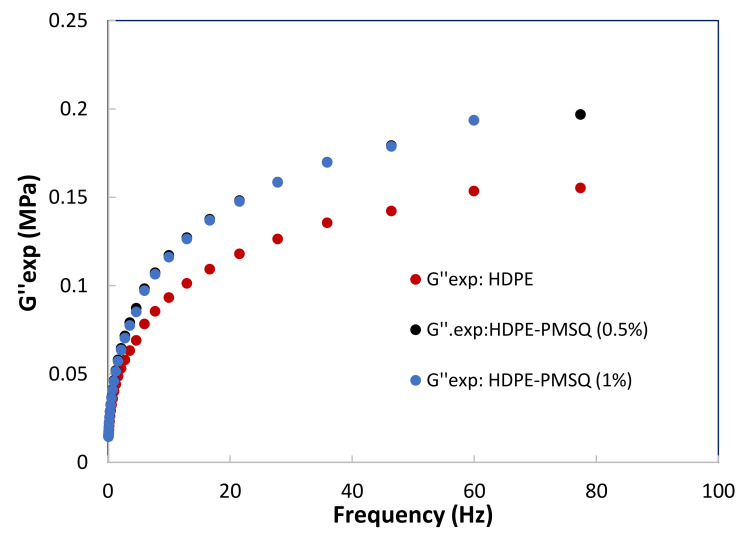
Experimental results of the loss moduli (G”) as a function of the PMSQ nanoparticles content.

The numerical simulation of thermoforming of thin parts such as drone blades requires an adequate description of the viscoelastic behavior of the composite materials as a function of both the temperature and the particles reinforcement concentration (PMSQ). In order to achieve this purpose, small amplitude oscillatory shear tests [17,18,19] were considered to determine the storage and loss moduli of the formulations as a function of frequency. The results are shown in Figure 2 and Figure 3 for HDPE reinforced with 0, 0.5 and 1.0% (*w*/*w*) PMSQ particles at 160 °C.

## 3. Numerical Modelling of the Blade’s Thermoforming Process

### 3.1. Characterization of HDPE-PMSQ’s Viscoelastic Behavior

This section is based on the data previously acquired on the samples of HDPE reinforced with PMSQ nanoparticles. These composite materials were assumed to be isotropic, homogeneous and isothermal for modelling purposes. Taking into account the numerous bibliographical references, Lodge’s integral viscoelastic model seems suitable to represent the viscoelastic behavior of HDPE and composites, based on sawdust and HDPE [20]. For this reason, we explored this model to adjust the viscoelastic response of HDPE-PMSQ.

In regard to many literature references, Lodge’s integral viscoelastic model—which is suitable for representing the behavior of HDPE and HDPE-based composites [20]—was applied to fit the viscoelastic response of the HDPE-PMSQ.

The Lodge constitutive model forms the basis of most integral viscoelastic models [17]. It is often used to describe the incompressibility behavior of thermoplastic polymers in their liquid state. However, Erchiqui et al. [18] have shown that this model can be used for semi-solid materials as well. According to this model, the knowledge of the material’s strain history is essential to determine the Cauchy stress tensor described by Equation (1):

(1) $$\sigma \left(t\right)=-p\left(t\right)I+\int_{-\infty }^{t}\sum_{k=1}^{n}\frac{g_{k}}{\tau_{k}}e^{\left(-\frac{t-\tau }{\tau_{k}}\right)}B(t,\tau )\;d\tau$$

where *p*, *I*, *B*, *t* and *τ* are the hydrostatic pressure, identity tensor, finger deformation tensor, time and relaxation time, respectively. The parameters *g_k_* and *τ_k_* are, respectively, the stiffness modulus and the relaxation time associated with the mode. The stress tensor is related to the deformation gradient tensor as shown in Equation (2):

(2) $$B\left(t,\tau \right)={\left[F^{T}\left(t,\tau \right)\cdot F\left(t,\tau \right)\right]}^{-1}.\;$$

There are several approaches, which combine experimental and numerical data to identify the rheological properties of the Lodge model. In this work, we consider the experimental data from previous studies undertaken using low-amplitude oscillatory shear tests on the HDPE-PMSQ which respectively provide the storage and loss moduli $G^{\prime }$ and $G^{\prime \prime }$. Moreover, the least squares method is used to minimize the discrepancies between the experimental and theoretical values during the identification of the relaxation spectrum for each type of HDPE-PMSQ. This method is described by reducing the objective function defined by Equation (3) where is the number of experimental data points:

(3) $$Z=\sum_{i=1}^{N}{\left[\frac{G_{i,exp}^{\prime }-G_{i,th}^{\prime }}{G_{i,exp}^{\prime }}\right]}^{2}+{\left[\frac{G_{i,exp}^{\prime \prime }-G_{i,th}^{\prime \prime }}{G_{i,exp}^{\prime \prime }}\right]}^{2}$$

The parameters $G_{i,exp}^{\prime }$ and $G_{i,exp}^{\prime \prime }$ represent the dynamic moduli from the experimental data while $G_{i,th}^{\prime }$. and $G_{i,th}^{\prime \prime }$. represent the theoretical values given by Equation (4) [20].

(4) $$G^{\prime }\left(\omega \right)=\sum_{i=1}^{N}\frac{g_{i}\tau_{i}^{2}\omega^{2}}{1+\tau_{i}^{2}\omega^{2}}\;\;\mathrm{and}\;\;\;G^{\prime \prime }\left(\omega \right)=\sum_{i=1}^{N}\frac{g_{i}\tau_{i}\omega }{1+\tau_{i}^{2}\omega^{2}}$$

**Figure 4:**
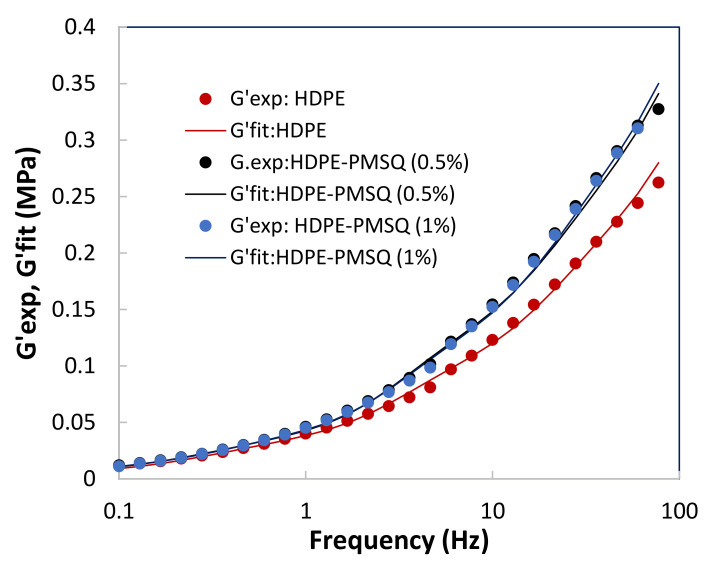
Results of the optimization with the experimental storage moduli G′ as a function of the PMSQ nanoparticles content.

**Figure 5:**
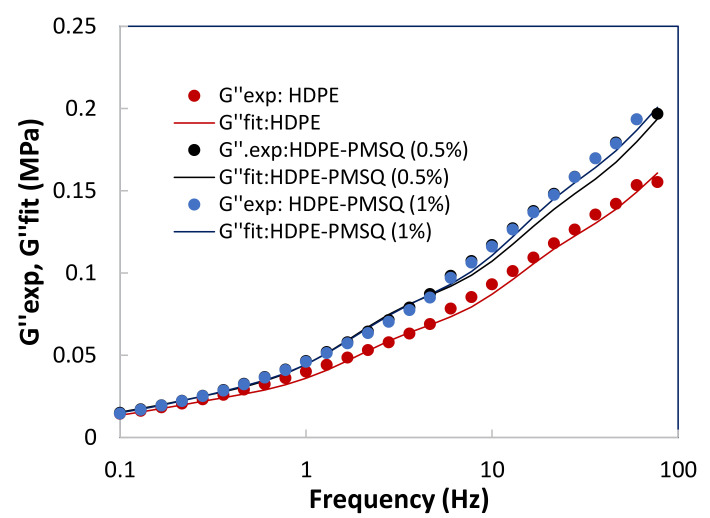
Results of the optimization with the experimental loss moduli G″ as a function of the PMSQ nanoparticles content.

The parameters *g_i_* is the stiffness constant and *τ_i_* is the relaxation time associated with the mode *i*, while *ω* is the frequency. The results obtained are given in Table 4. Figure 4 and Figure 5 show the results of the optimization represented by the dotted lines in comparison with those of the experiment represented by the points. 

### 3.2. Finite Element Formulations

In this section, the explicit dynamic finite element method with both space and time discretization, is used to simulate the formation of the composites part made of different concentrations of PMSQ nanoparticles and a thermoplastic matrix. The principle of virtual work is expressed on the undeformed configuration for the inertial effects and internal work [19].

#### 3.2.1. Finite Element Discretization 

Spatial and temporal discretization are both necessary for the virtual work due to the presence of the force of inertia. In the case of spatial discretization, the finite element method approach is considered [20]. However, for temporal discretization, the centered finite difference method, which is conditionally stable, is used. After summing up the contributions of all the elements, the inflation problem is then reduced to the following discrete system of equations [20]:

(5) $$M\;\ddot{u}=F_{ext}+F_{grav}-F_{int}$$

where *F_ext_*, *F_grav_* and *F_int_* are the global nodal external, body, and internal force vectors applied to the thermoplastic membrane. $\ddot{u}$ is the global nodal acceleration vector an *M* is the mass matrix. Using the diagonalization method of Equation (5), the matrix *M* is transformed into a diagonal matrix and each degree of freedom can be handled independently. Finally, using a centered finite difference scheme, Equation (5) can be rewritten as Equation (6) [16]:

(6) $$u_{i}\left(t+\Delta t\right)=\frac{\Delta t^{2}}{M_{ii}^{d}}\left(F_{i}^{ext.}\left(t\right)+F_{i}^{grav.}\left(t\right)-F_{i}^{int.}\left(t\right)\right)+2u_{i}\left(t\right)-u_{i}\left(t-\Delta t\right)$$

where $M_{ii}^{d}$ are the diagonal components of the *M^d^* matrix. For more details on the implementation of the GEF, we suggest that the reader refer to reference [18].

#### 3.2.2. Convergence Stability Criteria

The convergence behavior of the explicit dynamic finite element method for non-linear problems is controlled by the Courant–Friedrichs–Lewy criterion which stipulates that the time step must be smaller than the critical time steps ($\Delta t\leq t_{crit}$):

(7) $$\Delta t_{crt}=\varepsilon \frac{l}{c}$$

Here, *c* is the wave speed in the medium and *l* is the element size. The quantity *l*/*c* is the time required for a wave to propagate across an element of size *l*. The proportionality constant *ε* depends on the integration scheme use, for stability condition, *ε* satisfies 0 < *ε* < 1. The initial conditions are further required in order to solve the problem at hand. The displacement and velocity vectors at the initial time are assumed zero for the material forming process.

#### 3.2.3. Plane-Stress Assumption 

In order to successfully model and simulate the inflation phase of the composite polymeric membrane which is first heated and then blown, an accurate evaluation of the internal force is required. The relationship between deformations and stresses must be computed for each element. Using the plane-stress assumption along the incompressibility conditions of the thermoplastic material, it follows that the components of the Cauchy stress tensor satisfy the conditions as listed in Equation (8).

(8) $$\sigma_{13}=\sigma_{23}=\sigma_{31}=\sigma_{32}=\sigma_{33}=0$$

#### 3.2.4. Constitutive Equation 

In order to address the behavior of the isotropic thermoplastic composite, the Lodge constitutive model [17] is applied. In this model, the Cauchy stress tensor *σ*(*t*) is related, at time *t*, to the history of the Finger deformation tensor *B*(*t*,*τ*) by Equation (9):

(9) $$\sigma \left(t\right)=-p\left(t\right)I+\int_{-¥}^{t}\sum_{k}\frac{g_{k}}{\tau_{k}}e^{-\left(t-\tau \right)/\tau_{k}}B\left(\tau ,t\right)\;d\tau$$

where the finger tensor **B** is related to the right Cauchy Green deformation tensor **C** by Equation (10).

(10) $$B=C^{-1}={\left(F^{T}F\right)}^{-1}$$

**F** is the deformation gradient tensor, *p* is the hydrostatic pressure, while *g_k_* and *τ_k_* represent the stiffness moduli and relaxation time, respectively. The temperature dependence of these models is accounted for using the Williams–Landel–Ferry (WLF) equation [21].

#### 3.2.5. Van der Waals Equation of State and Pressure Loading 

In this study, it is considered that the external pressure induced by the airflow load is responsible for the blowing of the membrane. The pressure inside the thermoplastic-based composite membrane is closely related to the internal volume, according to the Redlich–Kwong gas equation of state given by Equation (11) [22].

(11) $$P\left(t\right)=\frac{n\left(t\right)RT_{gas}}{V\left(t\right)-nb\left(t\right)}-\frac{n^{2}\left(t\right)a}{V\left(t\right)\left(V\left(t\right)+n\left(t\right)b\right)\sqrt{T_{gas}}}$$

where *n*(*t*) is the number of gas moles introduced to inflate the thermoplastic composite membrane, *P*(*t*) is the internal pressure, *V*(*t*) is the volume occupied by the membrane at time *t*, *T_gas_* is the absolute gas temperature, and *R* is the universal gas constant (=8.3145 J·mol^−1^·K^−1^). “*a*” and “*b*” are constants evaluated from the critical state of the gas [22]:

(12) $$a=0.42748\frac{{\bar{R}}^{2}T_{c}^{2.5}}{p_{c}}\;and\;b=0.08664\frac{\bar{R}T_{c}}{p_{c}}$$

where *T_c_* and *p_c_* are the critical temperature and pressure of the gas, respectively. The critical temperature of a material is the temperature above which distinct liquid and gas phases do not exist. As the critical temperature is approached, the properties of the gas and liquid phases become the same, resulting in only one phase known as the supercritical fluid. Above the critical temperature, a liquid cannot be formed by an increase in pressure, but with enough pressure, a solid may be formed. The critical pressure is the vapor pressure at the critical temperature.

In this study, the assumptions used for the calculation of the dynamic pressure are:

(i) Gas temperature is assumed constant (*T_gas_*)
(ii) The Composite sheet temperature is assumed constant (*T_sheet_* = *T_gas_*)
(iii) At every moment, the pressure between the sheet and the mold is assumed constant
(iv) The contact between the composite sheet and the mold is assumed to be a sticky contact as the polymer cools and stiffens rapidly during the sheet/mold contact.

If *V*_0_ is used to represent an initial volume enclosing the membrane at the initial time *t*_0_ and containing a number *n*_0_ of gas moles while assuming that the forming process temperature is constant, the Redlich–Kwong equation of state is reduced to Equation (13):

(13) $$P_{0}=\frac{n_{0}RT_{gas}}{V_{0}-n_{0}b}-\frac{n_{0}^{2}a}{V_{0}\left(V_{0}+n_{0}b\right)\sqrt{T_{gas}}}$$

when *n*(*t*) moles of gas are introduced to inflate the thermoplastic composite membrane, *P*(*t*) is the internal pressure, and *V*_0_ + *V*(*t*) is the volume occupied by the membrane at time *t*, Equation (14) can be re-written as:

(14) $$\begin{array}{ll} \Delta P\left(t\right)=P\left(t\right)-P_{0} & =\left[\frac{\left(n\left(t\right)+n_{0}\right)RT_{gas}}{\left(V\left(t\right)+V_{0}\right)-\left(n\left(t\right)+n_{0}\right)b}-\frac{{\left(n\left(t\right)+n_{0}\right)}^{2}a}{\left(V\left(t\right)+V_{0}\right)\left(\left(V\left(t\right)+V_{0}\right)+\left(n\left(t\right)+n_{0}\right)b\right)\sqrt{T_{gas}}}\right]\\ & -\left[\frac{n_{0}RT_{gas}}{V_{0}-n_{0}b}-\frac{n_{0}^{2}a}{V_{0}\left(V_{0}+n_{0}b\right)\sqrt{T_{gas}}}\right] \end{array}$$

Equation (15) represents the time evolution of pressure inside the thermoplastic composite membrane. It is closely related to the evolution of the internal volume of the membrane under the thermodynamic state equation. In this case, the external virtual work is expressed in terms of a closed volume becomes Equation (15) that follows [22]:

(15) $$\delta W^{ext}=\Delta P\left(t\right)\;\delta V$$

The advantage of using a load expressed in terms of gas flow instead of pressure load is that it allows a natural exploration of the load-deformation curve without dealing with the instability associated with the classical pressure loading. The introduction of the constant pressure as a load force instead of the gas flow velocity in the finite element formulations leads to a divergence in computations for pressure values exceeding the critical point (beginning of the unstable segment of the load-deformation curve) [22,23].

#### 3.2.6. Work and Power for the Thermoforming of the Unmanned Aerial Vehicle (UAV) Blade 

The amount of energy required for the forming process is equivalent to the external mechanical work transferred by the external forces to the composite structure during the forming stage. This work is given in compact form as Equation (16).

(16) $$W^{ext}=\;{\left\{u^{n}\right\}}^{T}\times \left\{F_{ext}\right\}$$

where $\left\{F_{ext}\right\}$ is the global nodal external force vector applied to the thermoplastic composite membrane and $\;{\left\{u^{n}\right\}}^{T}$ is the associated global nodal displacement vector. The power associated with the global nodal external force vector is the amount of external work divided by the time interval required to form the polymeric part (energy per unit time transferred to the sheet during the forming stage):

(17) $$P^{ext}=\;\frac{W^{ext}}{t_{forming}}$$

The amount of energy and the power associated with the forming phase of a part determines the cost and feasibility of its manufacture for industrial purposes.

## 4. Thermoforming of a Thin National Advisory Committee for Aeronautics (NACA) Profile for UAVs

The dynamic finite element method outlined in the previous section was implemented in ThermoForm, the general purpose finite element code. This code was developed by the first author to study stresses and deformations occurring in thermoforming sheets as well as stretch blow molding problems. 

The nanocomposite studied was made of HDPE reinforced with 0, 0.5 and 1% (*w*/*w*) PMSQ nanoparticles labelled by HDPE-PMSQ1 and HDPE-PMSQ2, respectively.

**Figure 6:**
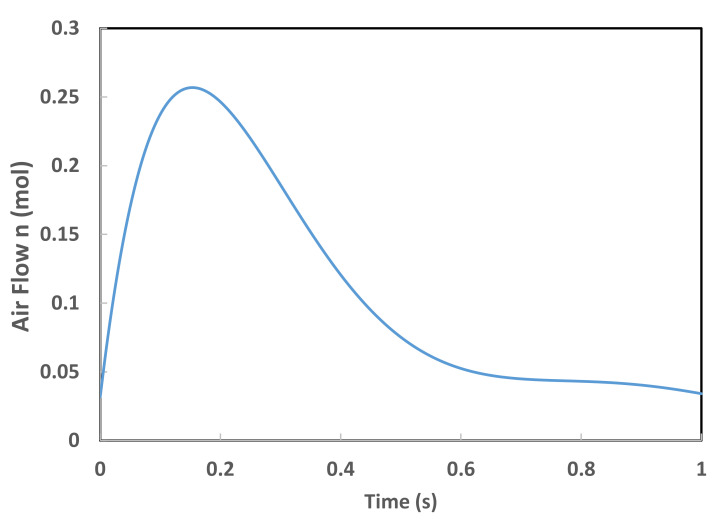
Airflow versus time.

**Figure 7:**
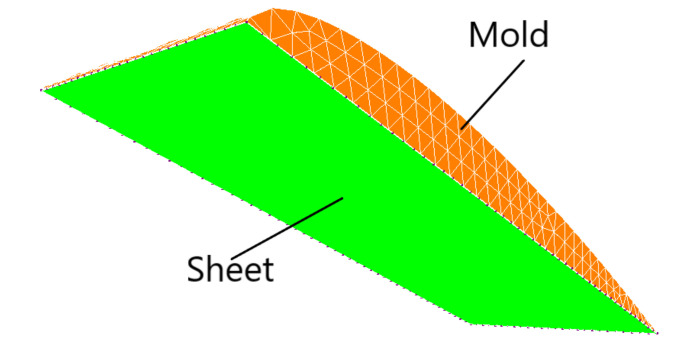
Geometries of the mold and the HDPE-PMSQ sheet.

The application considered was made by the thermoforming of a 25 cm by 10 cm rectangular membrane made of HDPE reinforced with PMSQ nanoparticles, with a load expressed in terms of non-linear airflow rate as shown in Figure 6. Its initial thickness h_0_ was 2.0 mm. The geometries of the mold of NACA (National Advisory Committee for Aeronautics) and the composite sheet discretized by triangular membrane elements as shown in Figure 7. The edges of the composite sheets are fixed for the modeling purpose. This means that the displacement of the nodes of the quarter edges of the sheet are zero. The material temperature was assumed to be constant at 16 °C. The rheological parameters of the Lodge behavior law are given in Table 4.

**Figure 8:**
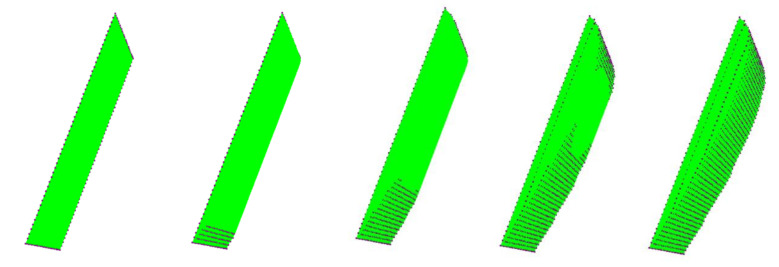
Evolution of the distribution of the contact nodes with the mold at the beginning, middle, and the end of the forming cycle.

Figure 8 illustrates the evolution of the nodes, which are in contact with the mold during forming process, represented by the black dots. 

**Figure 9:**
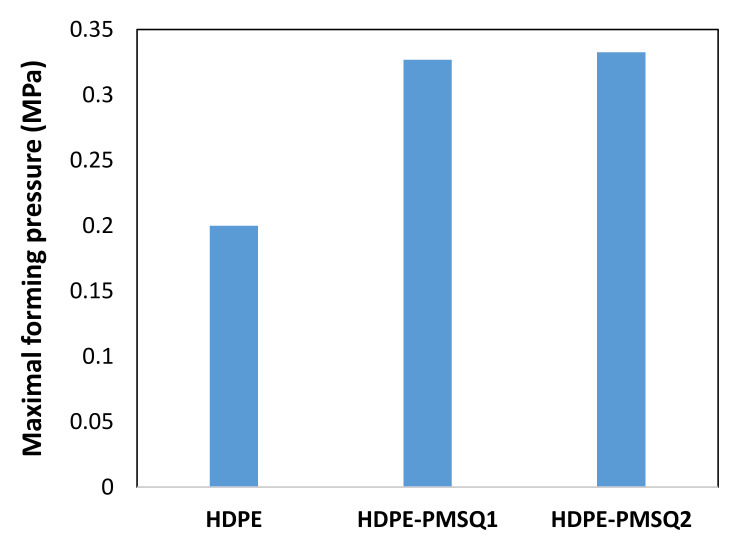
Maxima internal pressure forming.

According to Figure 9, the maximum internal pressure induced at the end of the shaping of the composite blade depends on the proportion of PMSQ nanoparticles in the composite. For HDPE-PMSQ2, the maximum pressure induced at the end of the thermoforming cycle is 0.3326 MPa compared to that used in the virgin HDPE matrix which is 0.2725 MPa. In the case of HDPE-PMSQ1, the maximum internal pressure is 0.3268 MPa. 

**Figure 10:**
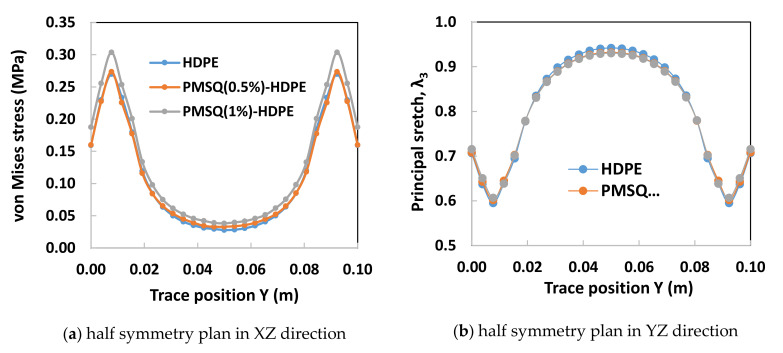
Von Mises stress on XZ and YZ half planes of symmetry of the blade.

**Figure 11:**
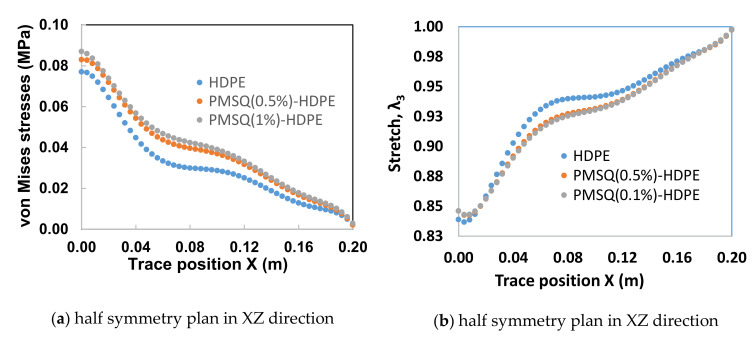
Main stretch (λ_3_) on the XZ and YZ half planes of symmetry.

When simulating the thermoforming of a thin part, it is important to predict the thickness and stress distribution in the molded part. In fact, the predictions of the residual stresses and dimensional stability of the final shape of the molded part is closely related to the estimated stresses. In addition, the effect of localized thinning of the deformed membrane is usually accompanied by an increase in the Cauchy stresses (or actual stresses). On each trace of the XZ and YZ half-planes of the thermoformed blade, the von Mises stress distributions and the main extensions as a function of the PMSQ nanoparticle content are shown in Figure 10 and Figure 11, respectively.

**Figure 12:**
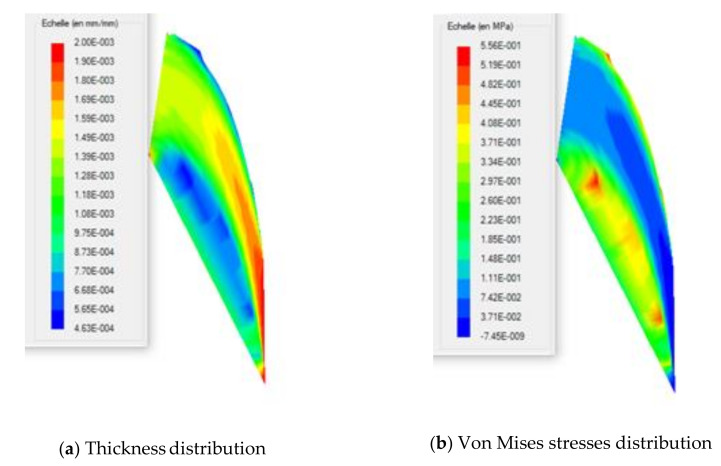
Distributions of the final thickness and the von Mises stresses in the thermoformed blade.

Critical values of von Mises stresses and induced thicknesses in the XZ and YZ half-planes of the blade are given in Table 5 and Table 6, respectively. The von Mises stress distribution on the XZ half-plane exhibits a maximum of 0.087 MPa for HDPE-PMSQ2 and the minimum of 0.077 MPa for virgin HDPE (See Table 4). The thicknesses associated to its critical stresses are respectively 1.686 mm for HDPE-MSQ2 and 1.674 mm for HDPE. These values are not identical in the YZ half-plane. Indeed, as shown in Table 5, the von Mises stress distribution on the YZ half-plane shows the maximum of 0.304 MPa for HDPE-PMSQ2 and the minimum of 0.270 MPa for virgin HDPE. The thicknesses associated to its critical stresses are respectively 1.213 mm for HDPE-MSQ2 and 1.190 mm for HDPE. Finally, the von Mises stress distribution and the localized thinning effect indicate that material failure due to large inflation-induced deformations is more likely to occur in the left tip area of the blade as shown in Figure 10b and Figure 11b, respectively. These observations show that the von Mises stresses induced in the HDPE-PMSQ1 and HDPE-PMSQ2 composite blades are significantly greater than those introduced in the PHDE. However, the pressures and times required to manufacture the blades remain comparable, hence the potential of PMSQ-HDPE composites to compete with HDPE. Figure 12a,b show, respectively, views of the von Mises stress distribution and the main extensions in the final shape of the thermoformed part for the HDPE-PMSQ2 composite.

**Figure 13:**
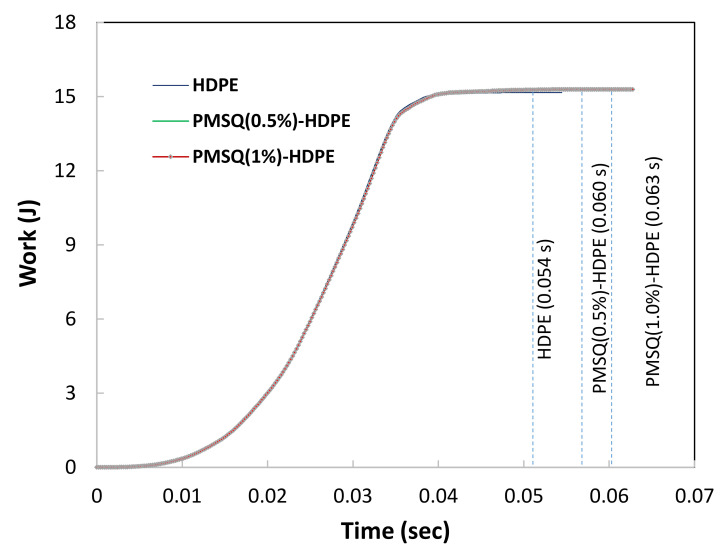
Work done by the gas at the end of the forming cycle for HDPE, HDPE-PMSQ1 and HDPE-PMSQ2.

**Figure 14:**
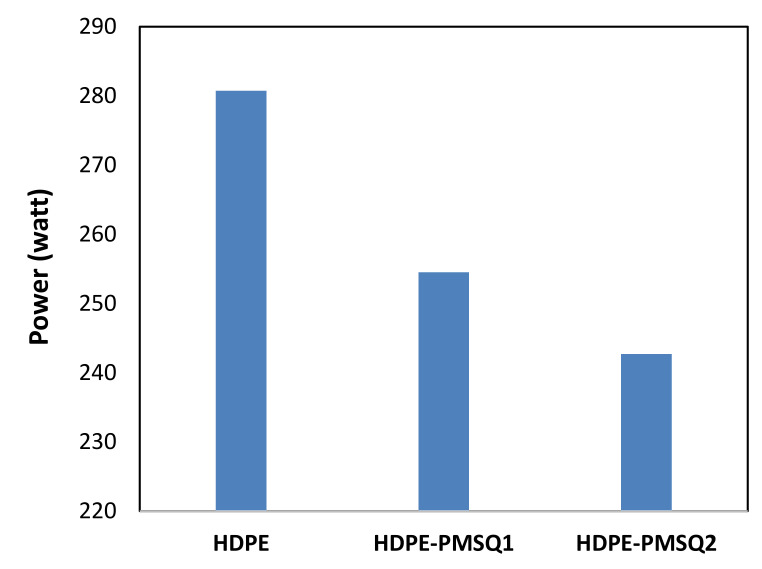
Values of the work at the end of the forming cycle for different formulations of HDPE reinforced with PMSQ.

Figure 13 shows the work required over time for thermoforming HDPE, HDPE-PMSQ1 and HDPE-PMSQ2. This work takes the values of 15.16, 15.27 and 15.29 joules for virgin HPDE, HDPE-PMSQ1 and HPDE-PMSQ2, respectively. From a cost-benefit standpoint, the forming time is also an important issue especially for mass production. The results shown in Figure 13 show that the forming time increases with the amount of PMSQ nanoparticles contained in the composite material. The time required for the forming of the composite HDPE-PMSQ1 is 1.75 times longer than the time needed for virgin HPDE. Therefore, it appears that, based on the forming work and the duration of the process, the power required to thermoform the blade (see Figure 14) is maximum for HDPE (270 watt) and almost similar for HDPE-PMSQ1 (244 watt) and HDPE-PMSQ2 (242 watt). Moreover, it is important to note that these results were obtained without considering the energy dissipated by frictional contact between the mold and the polymer/composite materials.

According to the same figure, it appears from the simulation that the forming time increases with the PMSQ nanoparticles content. In this regard, the forming time was 0.056 s for virgin HDPE and 0.063 s for HDPE-PMSQ2. This is in line with previous results where PMSQ nanoparticles have made the composite slightly more resistant to deformation.

In light of the results given above, the following remarks can be formulated:

− The numerical results show the potential use of HDPE-PMSQ composites for the manufacture of UAV blades by thermoforming.
− The moduli of elasticity of HDPE-PMSQ composites (1064 MPa in the case of 0.5% PMSQ and 1115 MPa in the case of 1.0% PMSQ) are more interesting than those of virgin PHDE which is 1031 MP.
− The viscoelastic behavior of HDPE-PMSQ composites shows little influence on the thickness and stress distributions in the final product.
− The work and power required to form the UAV blade for the virgin polymer and composites are almost similar.
− The thickness and von Mises stresses obtained from the different composites are not similar, especially the stress distribution.
− The amount of material in the central area of the UAV blade increases slightly compared to the virgin polymer.

The numerical results show the potential use of HDPE-PMSQ composites for the manufacture of a UAV blade by thermoforming. Moreover, the choice to use the finite element method with a pressure load, which is derived from a thermodynamic law, is judicious for the integrated analysis of thermoforming of thin parts. 

## 5. Conclusions

A family of composites based on high density polyethylene and PMSQ nanoparticles was developed, characterized and numerically analyzed for their potential use in the manufacture of small UAV blades, through the thermoforming process. The viscoelastic behavior of the Lodge model was considered. Moreover, the Lagrangian equation together with an assumption of membrane theory was used in the finite element implementation. This study shows that:

(i) The constitutive behavior of HDPE reinforced with PMSQ nanoparticles (with mass concentrations of 0.5 and 1%) has little influence on the final thickness and stress distribution in the blade;
(ii) The time required to thermoform a composite part based on HDPE and PMSQ (with mass concentrations of 0.5 and 1%) is practically unchanged;
(iii) The energy used for the thermoforming of the composite blade is practically the same for the mass concentrations of 0.5 and 1% PMSQ;
(iv) The numerical results show the potential use of composites for the manufacture of drone blades by thermoforming.

## Figures and Tables

**Table 1:** Ladder polymethylsilsesquioxane (PMSQ) nanoparticles properties [4].

Specific Area BET (m^2^/g)	Density (g/cm^3^)	Size (nm)	Contact Angle (Degree)
0.1154	1.42	15–20	148 ± 3

**Table 2:** Tensile mechanical properties of high-density polyethylene (HDPE)-PMSQ nanocomposites [16].

% PMSQ-HDPE	Elastic Modulus (MPa)	Yield Stress (MPa)	Elongation at Beak (%)
0.0%	1031 ± 26	26.8 ± 0.2	39.2 ± 2.3
0.5%	1064 ± 60	27.9 ± 0.3	47.2 ± 3.1
1.0%	1115 ± 54	30.1 ± 0.1	41.1 ± 2.3

**Table 3:** Differential scanning calorimetry (DSC) melting temperature (T), crystallinity temperature (T) and crystallinity value o HDPE-PMSQ nanocomposites [16].

Parameters	HDPE	HDPE-0.5%	HDPE-1%
T_m_/K	404.5	404.5	405.5
T_c_/K	390.8	390.5	391.9
ΔH/J g^−1^ (melting)	189.8	190.9	194.3
X_c_%	64.9	65.0	66.4

**Table 4:** Stiffness modulus and relaxation time for the composites at T = 160 °C.

*τ_k_* (s)	HDPE	HDPE-PMSQ (0.5%)	HDPE-PMSQ (1%)
*τ_k_* (s)	*g_k_* (MPa)	*g_k_* (MPa)	*g_k_* (MPa)
0.01249	0.314674	0.378765	0.389422
0.08746	0.123293	0.147477	0.158700
0.61220	0.069532	0.093907	0.088718
4.28570	0.016151	0.014978	0.019317
10.0000	0.012357	0.014191	0.010610
50.0000	0.006604	0.007997	0.008955

**Table 5:** Critical values of the von Mises stresses and thickness in the XZ half-plane.

% PMSQ	σ_crit_ (Mpa)	h_cr_ (mm)
0%	0.077	1.674
0.5%	0.083	1.686
1.0%	0.087	1.686

**Table 6:** Critical values of the von Mises stresses and thickness in the YZ half-plane.

% PMSQ	σ_crit_ (Mpa)	h_cr_ (mm)
0%	0.270	1.190
0.5%	0.274	1.204
1.0%	0.304	1.213

## Data Availability

The study did not report any data.
